# Building trusting relationships to support implementation: A proposed theoretical model

**DOI:** 10.3389/frhs.2022.894599

**Published:** 2022-09-23

**Authors:** Allison Metz, Todd Jensen, Amanda Farley, Annette Boaz, Leah Bartley, Melissa Villodas

**Affiliations:** ^1^School of Social Work, University of North Carolina at Chapel Hill, Chapel Hill, NC, United States; ^2^London School of Hygiene and Tropical Medicine, London, United Kingdom

**Keywords:** implementation science, implementation practice, trust, relationships, theory of change

## Abstract

This paper presents a theory of change that articulates (a) proposed strategies for building trust among implementation stakeholders and (b) the theoretical linkages between trusting relationships and implementation outcomes. The theory of change describes how trusting relationships cultivate increases in motivation, capability, and opportunity for supporting implementation among implementation stakeholders, with implications for commitment and resilience for sustained implementation, and ultimately, positive implementation outcomes. Recommendations related to the measurement of key constructs in the theory of change are provided. The paper highlights how the development of a testable causal model on trusting relationships and implementation outcomes can provide a bridge between implementation research and implementation practice.

## Introduction

The implementation of effective policies, practices, and approaches is critical to optimizing patient care in the context of health services. To increase the generalizability of findings from implementation research to implementation practice, the field of implementation science has begun to call for greater conceptual clarity on important aspects of implementation (1). This call for greater conceptual clarity may be due, in part, to growing discussions in the field of implementation science on the divide between implementation research and implementation practice (2). Implementation research seeks to understand the approaches that work best to translate research to the real world, whereas implementation practice seeks to apply and adapt these approaches in different contexts to achieve outcomes (3).

Trust is an example of an implementation construct that needs to be further operationalized so that implementation researchers can study its role in implementation and implementation practitioners can test strategies to foster and deepen trust among implementation stakeholders. Common definitions of interpersonal trust appeal to McAllister's [(4), p.25] articulation as follows: “the extent to which a person is confident in and willing to act on the basis of the words, actions, and decisions of another.” Trusting relationships are centered in vulnerability where the beliefs or expectations of individuals in the relationship are that actions will cause no harm and will provide benefit (5–8).

Although trusting relationships are commonly described as important by implementation stakeholders involved in leading implementation efforts (9–11), few studies have explored this topic in depth (12–14). The dearth of research in this area limits our theoretical and practical understanding of how trusting relationships among implementation stakeholders can be effectively built and why they are important. Implementation stakeholders refer to all individuals and groups who have an interest in the implementation result and, therefore, require authentic involvement in the implementation process (15). In this paper, we describe how professionals providing implementation support—referred to as implementation support practitioners (ISPs) (16, 17)–can build trust with and among implementation stakeholders, consequently leading to improved implementation results. In a recent study, highly experienced ISPs emphasized that high-quality relationships among implementation stakeholders was a—if not *the*—critical factor for achieving implementation results (2).

Conceptual clarity regarding the role of trusting relationships in implementation will enable the development of research designs and measures that could aid in answering important research questions, such has how trust moderates associations between implementation strategies and implementation outcomes. Consistent terminology and definitions are needed to describe the relational aspects of implementation. Research on trust can also produce generalizable knowledge related to trust-building among implementers, thereby creating a virtuous learning cycle between implementation researchers and ISPs.

This paper will present a theory of change that highlights promising strategies for building trust among implementation stakeholders. The theory of change also explicates how trusting relationships promote motivation, capability, and opportunity for supporting implementation among implementation stakeholders, with implications for commitment to and resilience for sustained implementation, and ultimately, positive implementation outcomes. We will first describe the role of trust in implementation practice, followed by a description of the theoretical models that have informed the proposed theory of change and the assumptions that underly the connections between trust and implementation outcomes. We will also provide examples of how trust and other key constructs in the theory of change can be measured and how the development of this testable causal model can provide a bridge between implementation research and implementation practice.

### Conceptualizing trust

Historically, efforts to understand trust have incorporated rational choice perspectives that emphasize self-interest as a primary motivating force; however, these perspectives justly have been criticized for failing to fully account for the role of perceptions, attributions, and affective processes associated with relational trust-building (4, 7, 18–23). The theory of change describes both relational and technical strategies for building trust. Whereas technical strategies are grounded in intrapersonal and cognitive dimensions of trust, relational strategies are grounded in interpersonal and affective dimensions of trust (4, 24). *Intrapersonal trust* refers to the belief that a team member or stakeholder is reliable, competent, and committed to the goals of the implementation team. *Interpersonal trust* refers to the perception of implementation team members and stakeholders that they are in a collaborative and reciprocal relationship in pursuit of the same aims (17).

Similarly, McAllister (4) highlights key differences between *cognitive* and *affective* dimensions of trust. Cognition-based trust reflects individuals' cognitive evaluations of the reliability, integrity, and competence of others; whereas affect-based trust reflects individuals' feelings of emotional involvement and others' genuine care and concern for their welfare (8). Thus, trustors often endeavor to observe the following: (a) whether trustees' behavior indicates that they are competent to perform according to expectations, and (b) whether trustees' behavior conveys an intention to invest in and maintain a working relationship (7). Many researchers agree that trust is cultivated primarily by a combination of these cognitive/intrapersonal and affective/interpersonal factors (6).

### Trust in implementation teams

Implementation efforts often rely on implementation teams (25). An implementation team is a group of stakeholders (e.g., program administrators, practitioners, program developers, funders, recipients of program services, community members) that oversees, manages, and is accountable for performing key functions with respect to the selection, implementation, and continuous improvement of a selected intervention (25). Implementation teams are a group with a common goal and are jointly responsible for ensuring completion of necessary tasks throughout all stages of the implementation process. Implementation teams offer a valuable alternative to “solo hero” models of implementation, whereby one or more individual leaders operate in siloes and struggle to effectively influence all necessary stakeholders in the context of an implementation effort. Higher levels of interpersonal trust, particularly in the context of teams, has been linked to higher levels of team satisfaction (26, 27), proactive idea implementation and problem-solving (28), information sharing (29, 30), team learning (31), team-member autonomy and task interdependence (32), affective commitment (27), organizational commitment (33), productivity (33), cooperation (34), and team performance (8, 35–38).

Evidence linking trust with team cooperation and team performance is especially compelling, given robust meta-analytic studies that have been conducted on these topics. Specifically, a meta-analysis of 212 individual studies assessing the association between trust and cooperation yielded a small-to-moderate average effect size [*r* = 0.26; (34)]. Another meta-analysis of 112 independent studies (including over 7,700 teams) yielded an above-average overall effect size (ρ = 0.30) linking intrateam trust and team performance—defined as the extent to which a team accomplishes its goal or mission (8). Taken together, the literature highlights numerous positive individual- and team-level outcomes associated with interpersonal trust.

### Trust in implementation practice

There is growing interest in the experiences of ISPs, including the strategies they use to support implementation and the skills required to use these strategies (39). Emerging competencies for this workforce; identified through research on the knowledge, skills, and attitudes of ISPs; uniformly identify building trusting relationships as a critical skill for progressing implementation (17, 40).

Furthermore, experienced ISPs have amplified the need to focus on relational issues in implementation and have identified trusting relationships as a critical aspect of implementation (16, 17). In general, the field of implementation is more routinely acknowledging the wide range of skills and attributes, both technical and relational, needed to effectively, and resiliently, provide implementation support (16, 41–43). Yet, trust-building is not explicitly named as an implementation strategy (44) and is not included in commonly used implementation frameworks.

Implementation strategies refer to “the methods or techniques used to enhance the adoption, implementation, and sustainability of a clinical program or practice” (45). Implementation strategies are used to affect change on specific implementation outcomes described by Proctor et al. as acceptability, adoption, fidelity, reach, and sustainability (45). Trust-building can be considered an implementation strategy in its own right. That is, trust can directly contribute to desirable implementation outcomes. Trust can also be conceptualized as a moderator of associations between commonly applied implementation strategies and implementation outcomes, magnifying the impact of any implementation strategy on implementation outcomes.

In the context of our proposed theory of change, we foreground trust-building as an implementation strategy. We also acknowledge that when trust is developed, trust can positively moderate the effects of other implementation strategies (46). Implementation strategies in existing taxonomies (e.g., Expert Recommendations for Implementing Change [ERIC] compilation) (44) often represent broad “labels” for implementation activities that leave ample room for further operationalization and tailoring by ISPs working in the often-complex settings of routine practice and policy implementation. The experiences of ISPs can help to further operationalize what it takes for implementation strategies to effectively contribute to implementation progress and outcomes.

For example, recent studies on the role of ISPs point to the relational processes and affective experiences of providing implementation support (47). In a recent study of evidence use (2), the importance of trusting relationships to enable successful implementation and sustained evidence use emerged as a central theme through interviews conducted with experienced ISPs. Study participants included professionals who support the use of evidence-based practices in child and family services. Participants described how they pivoted away from the use of specific implementation frameworks or methods, focusing more on developing trusting relationships and building teams in order to achieve implementation outcomes.

Additionally, in a recent study where experienced ISPs were interviewed (2), almost all participants reflected on a transformation in the way they provide implementation support, moving from didactic trainings to participatory models to co-creation. The majority of participants noted a current and desired state of implementation support focused on trusting relationships, driven by community data, and centered in co-creative approaches where both intervention and implementation strategies are co-designed with community members.

In summary, growing evidence highlights trusting relationships as a critical element of effective implementation, with important implications for efforts to bring to scale effective policies, practices, and approaches in the context of health services and other related service delivery settings. Valuable opportunities remain to develop theoretical models to guide future research and enrich our understanding of relational elements that optimize implementation efforts. The purpose of this paper is to provide conceptual clarity on the role of trusting relationships in implementation, which can strengthen the connection between implementation research and implementation practice. In this paper, we present a theoretical model on how trusting relationships ultimately contribute to implementation outcomes. This theoretical model integrates relational cohesion theory (48, 49), relational cultural theory (50), and implementation research findings related to mechanisms of change (51).

### Theoretical underpinning of trust-building

Theories of change are critical for conducting relevant implementation research. A theory of change outlines the predicted causal linkages between the activities conducted and the expected short-term, intermediate, and long-term outcomes for the population of interest. Ideally, a theory of change will detail implementation researchers' assumptions about how and why they expect a desired change in implementation outcomes to occur in a particular context (52). The proposed theoretical model is based on findings from our previous research on trust and evidence use, which demonstrates that building trusting relationships and addressing power differentials among stakeholders may be more important than the selection of specific implementation strategies for achieving implementation outcomes (17). The theoretical model articulates (a) underlying assumptions about the competencies needed to build trusting relationships, (b) the role trusting relationships play in behavior change (16, 53), and (c) how changes in individual and team behavior can contribute to implementation efforts in the context of health services and other related service delivery settings.

#### Relational cohesion theory

Relational cohesion theory offers an important theoretical basis for connecting trusting relationships to successful implementation. Relational cohesion is defined as the perception by individuals in an exchange relation that *their relationship is a unifying element or force in the social situation* (48, 49). Such perceptions lead to higher levels of commitment and collectively oriented behavior. Relational cohesion theory emphasizes how relationships that emerge from positive affective experiences are valuable in and of themselves and contribute to trusting relationships and increased resilience and commitment in the face of challenges. Relational cohesion theory is aligned with cultural exchange theory (54), and with literature on lessons learned from implementation science on the role of partnerships and relationships. Cultural exchange relies heavily on interpersonal processes that require the development and nurturance of reciprocal perceived trust (55, 56). Palinkas et al. (57) describe cultural elements of successful partnerships including flexibility and sensitivity to the needs of individuals in the partnership, openness and honesty associated with building and maintaining trust, and humility and tolerance in service to mutualism and shared understanding of the work.

Relational cohesion theory also seeks to explain the conditions under which positive emotions are experienced within the exchange relation; it can be used to explain the conditions under which instrumental exchanges of implementation support become more affective, emotional, and meaningful to stakeholders at the implementing site and what type of activities lead to trust among implementation stakeholders.

#### Relational cultural theory

Relational cultural theory (50) offers additional insights about the types of strategies needed to promote positive affect and increased resilience and commitment. Relational cohesion theory also highlights the role of empathy in supporting the growth-promoting relationships needed for implementation. Specifically, relational cultural theory posits that the ability to understand the perspective of others increases a sense of mutual interdependence and leads to a positive affective response by individuals engaged in a relationship (42), which is relevant to fostering trust on implementation teams. The underlying assumptions of relational cultural theory highlight the role of empathy in producing positive relationships. These theoretical assumptions are also aligned with emerging research findings related to the role of professionals who support evidence use. For example, Metz et al. (17) identified *empathy, curiosity, commitment, methodical, and transdisciplinary* as core principles related to providing implementation support, with empathy having achieved the highest level of agreement among professionals surveyed about how they approach their work to support evidence use. Findings also highlighted relational strategies, including empathy-driven exchanges, open communication, and demonstrations of authenticity and vulnerability as critical for building trusting relationships. Relatedly, Bührmann et al. (40) identified seven particular attitudes or orientations for supporting implementation: *professional, motivating, empathetic, collaborative, authentic, flexible and creative, and honest*. Also relevant are findings from Metz and Bartley (12) where bi-directional communication among implementation stakeholders was a contributor to effective and sustainable implementation of evidence-based practices in a New York City's public child welfare system.

Although relational cohesion theory highlights the affective experiences of those involved in the exchange, it relies on technical strategies (e.g., frequent interactions, provision of expertise) to achieve the positive emotional experience and does not explicitly include relational strategies grounded in the mutuality of the exchange. Thus, pairing relational cohesion theory with relational cultural theory (50) presents a fuller picture of the types of strategies needed to promote positive affect and increased resilience and commitment. Moreover, many scholars favor viewing interpersonal trust-building as a dynamic, transactional, and interactive process that unfolds over time and across numerous interactions (7). Both relational signaling theory (7) and costly signaling theory (58) emphasize this conceptualization, highlighting how individuals seeking to build trust must continually evidence to each other their trustworthiness.

## Theory of change

The proposed theory of change posits a series of action steps for ISPs that will lead to increased trust between them and implementation stakeholders and increased trust among implementation stakeholders, leading to positive and sustainable implementation outcomes. The proposed theory of change outlines the starting point for trust-building as addressing power differentials among implementation team members and stakeholders through co-creation and humility. This starting point aligns with the assumption of relational cohesion theory that productive exchanges occur when two or more people seek to jointly produce benefits they cannot achieve alone (49). Inherent in this assumption is that entering the implementation space with humility and a commitment to co-creation will be critical for building the trusting relationships needed for successful implementation and evidence use. The proposed theory of change also posits that, after addressing power differentials, a shared goal for the use of evidence can be established.

### Relational and technical strategies for trust-building

The proposed theory of change includes two core mechanisms of trust-building: relational strategies and technical strategies. *Relational strategies* are defined as strategies undertaken to build trust through strengthening the quality, mutuality, and reciprocity of interactions among team members and implementation stakeholders. *Technical strategies* are defined as strategies undertaken to build trust through demonstrating the knowledge, reliability, and competency to support the goals of the team. Below we describe the assumptions of our theoretical model's focus on relational and technical strategies for building trust and explore implications of this model for implementation research and practice. Examples of how ISPs may use relational and technical strategies to build trust with and among implementation stakeholders are featured in Table 1.

**Table 1:** Examples of activities for trust building strategies.

**Relational Strategies**	**Examples for Implementation Support Practitioners**
Vulnerability	Model comfort with uncertainty amid implementation challenges; ask questions; ask for support from implementation stakeholders
Authenticity	Encourage implementation stakeholders to share their perspectives openly and honestly: support implementation stakeholders to understand the values and beliefs of other implementation stakeholders
Bi-directional communication	Support feedback loops among implementation stakeholders so that implementation decisions and reactions to those decisions are shared back and forth
Co-learning	Provide opportunities for all implementation stakeholders to describe their expertise and experience so that stakeholders can learn from each other; value different types of expertise and experience that individuals may bring to the implementation effort
Empathy-driven exchanges	Support an implementation stakeholder to understand the perspective of another stakeholder; highlight areas of shared understanding and common goals
**Technical Strategies**	**Examples for Implementation Support Practitioners**
Frequent interactions	Develop standing implementation meeting schedules that emphasize frequency over duration
Responsiveness	Acknowledge requests for support from implementation stakeholders and respond to requests as quickly as possible
Demonstration of expertise	Share accurate and credible information in a timely manner with all implementation stakeholders
Achievement of quick wins	Celebrate early signs of implementation progress and share progress widely with implementation stakeholders

#### Relational strategies

Implementation teams involve interdependence; thus, team members depend on each other in various ways to achieve implementation outcomes. The theory of change highlights five relational strategies grounded in relational cultural theory and cultural exchange theory that demonstrate promise for fostering trust among implementation stakeholders. These include: (1) showing *vulnerability* (i.e., comfort in uncertainty, risk, and emotional exposure) (59, 60); (2) approaching interactions with *authenticity* (i.e., openly, honestly, and in alignment with values) (12); (3) engaging in *co-learning* (61); (4) engaging in *empathy-driven exchanges* (62); and (5) using *bi-directional communication* (24). These relational strategies are hypothesized to contribute to positive affective responses, perceived value-add, predictability, and a safe and secure learning environment, which will promote trusting relationships among team members. A common set of skills for employing all five relational strategies include the ability to actively listen, to offer free attention, and to suspend judgement (63).

Vulnerability, the first relational strategy highlighted in the theory of change, is thought to be at the center of trust-building. Indeed, the willingness to take risks is common to situations that require trust. Trust requires the willingness of a person or group to be vulnerable to the actions of another person or group, with the expectation that the other(s) will perform a particular action important to the trustor (64). Trust is the cornerstone for effective implementation teams. Trust engenders faith that partners can rely on each other to come through on agreements and to understand—and even anticipate—each other's needs and interests (65).

Studies on emotional acknowledgment demonstrate how authentic and bi-directional communication can foster interpersonal trust. Emotional acknowledgment refers to the verbal communication by which one implementation stakeholder signals recognition of another implementation stakeholder's emotional display. Such emotional acknowledgment has been demonstrated to foster interpersonal trust (58). Research in this area is rooted in costly signaling theory (66) and suggests when one person (e.g., an implementation team member or implementation stakeholder) emotionally acknowledges another person, it signals that the person acknowledging the emotion is willing to allocate time and resources to the person expressing the emotion.

Research on emotional acknowledgment also supports the use of specific relational strategies for trust-building, including authenticity and bi-directional communication, as well as the technical strategy of responsiveness. When implementation stakeholders use these types of strategies with others, they imply a willingness to use resources in the future that will attend to the needs of other implementation stakeholders, thereby fostering trust. The bi-directionality of communication is key because communication rooted in emotion requires sense-making from both parties. In the case of implementation teams, all team members will need to engage in sense-making to see how other team members feel about specific implementation decisions the team will need to make.

Metz et al. (17, 61) describe how co-learning also contributes to trust. They explain how implementation stakeholders must communicate and listen for the purpose of mutual understanding and the collaborative integration of different perspectives and types of knowledge. As implementation stakeholders engage in co-learning processes, they negotiate and build trust and respect for all perspectives, including those that may be at risk of being excluded from dialogue because of race, ethnicity, language, or status.

Relational cultural theory conceptualizes empathy as mutual, interactive, and humanist, serving as the foundation for growth-promoting relationships. Professionals supporting implementation often describe empathy as foundational for developing trusting relationships, which aligns with how they describe their day-to-day activities building affiliation, making personal connections, and recognizing themselves as outsiders. Metz et al. (61) have described the ways leaders and staff can demonstrate empathy, including affectively attuning to stakeholders at the implementing site, balancing flexible boundaries with role clarity, demonstrating comfort in a relational context, and recognizing the impact all stakeholders have on implementation activities and decision-making.

#### Technical strategies

The theory of change also highlights technical strategies, grounded in structures and processes that can support implementation teams in achieving results. These include: (1) supporting *frequent interactions* that relational cohesion theory posits are needed for successful exchanges; (2) demonstrating a high level of *responsiveness* to requests; (3) demonstrating *expertise* that can help the team achieve results (24); and (4) planning for and achieving *quick wins* (67) in service to longer-term goals.

Research demonstrates that trust can be brought about through frequent and informal opportunities for contact and exchange (68), enabling individuals to engage in the risk taking, learning, and behavior change required to support implementation efforts (69). Frequent interactions can ensure that information is readily exchanged, making it possible for team members and implementation stakeholders to influence implementation decisions, thereby garnering trust in the process (70). Frequent interactions can reduce uncertainty in the implementation process and expand the amount and type of information exchanged (49). Frequent interactions can also enhance team member satisfaction with exchanges and strengthen relationships (71, 72).

Related to frequent interactions, responsiveness can promote successful exchanges among team members, leading to more “asks” from those involved in the exchange, reinforcing the positive experiences, and producing satisfaction and stronger relationships, and eventually trust. Responsiveness demonstrates flexibility. Flexibility illustrated at intrapersonal, organizational, and initiative levels can suggest that team members are prepared to respond to shifts in the work as they emerge. These qualities demonstrate sensitivity to both individual members of the team and the collective, helping to build and maintain trust (73).

Demonstrating expertise and credibility also cultivates intrapersonal trust, ensuring that individual team members share information and show behaviors that indicate reliability, competency, and commitment to the goals of the team. Responsiveness and credibility allow for team members to constantly learn from and teach one another. Organizations committed to learning are more likely to have successful partnerships in service to implementation (74, 75).

Increased credibility and perceived trustworthiness of team members can also be fostered through the attainment of quick wins. A dynamic, bi-directional relationship exists between trust and team performance, with higher levels of trust impacting team performance (8) and past team performance influencing trust within teams (76). Intentionally planning for early successes and celebrating those quick wins are critical to building trust among team members and, in turn, impacting future performance (76, 77).

### Reinforcing mechanisms of relational and technical strategies and trusting relationships

As noted earlier, ISPs often emphasize high-quality relationships among implementation stakeholders as the most critical factor for achieving implementation results (46). Indeed, experienced ISPs have described how trust between themselves and stakeholders, as well as among key stakeholders, was foundational for successful implementation and the sustainability of evidence-based and evidence-informed programs and practices. Trusting relationships were described as “important,” “critical,” “essential,” and “foundational” for fostering an environment in which change efforts were optimized and sustained. Participants also emphasized how demonstrating authenticity, vulnerability, and empathy both builds trust and serves as evidence of trusting relationships. The theory of change presented in this paper highlights the cyclical relationship between the technical and relational strategies overviewed and trusting relationships, whereby strategies to cultivate trusting relationships will become more pronounced as relationships become more trusting.

**Figure 1:**
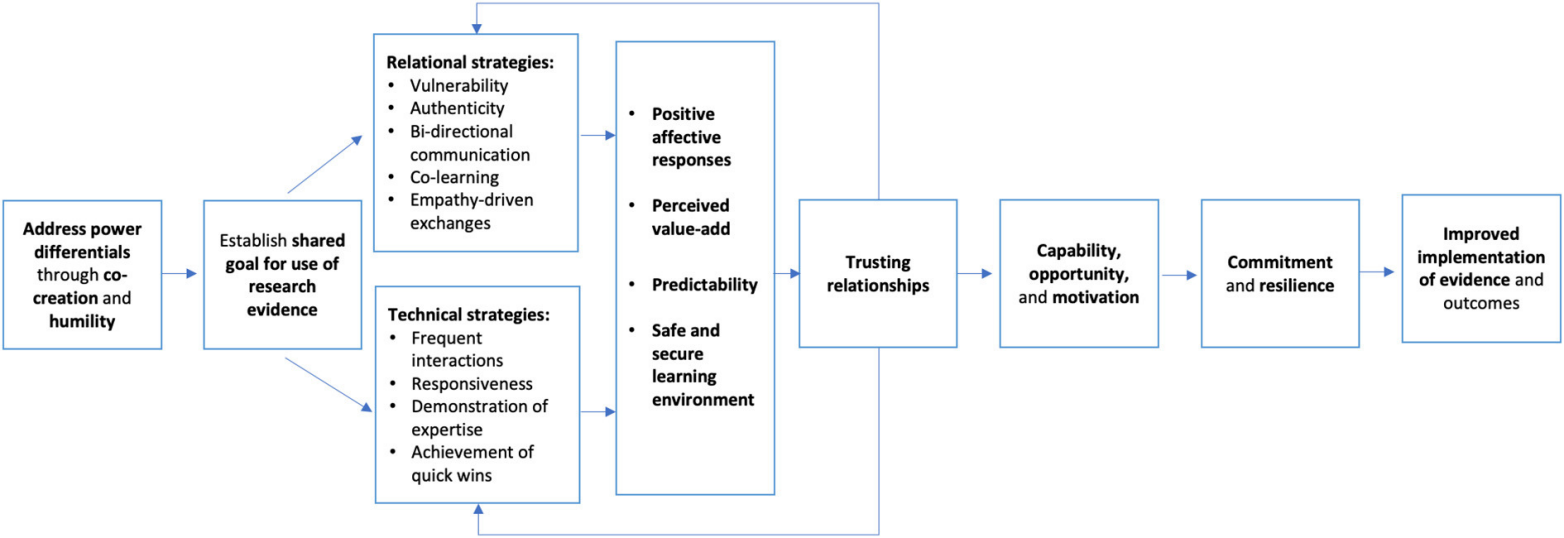
Theoretical model for trusting relationships and implementation.

The theory of change explicates how relational and technical strategies contribute to changes in behavior among implementation team members that promote positive implementation outcomes (see Figure 1 for a full visualization of the theory of change). Achieving these changes and the reinforcing cycle described above assumes that implementation team members have the capacity to use these strategies. A testable question is whether the skills needed to leverage relational strategies and build trust can be taught to and cultivated among implementation team members. Research in other fields such as business and leadership shows promise for increasing relationship-building and empathy in team leaders. For example, team leaders can use on-the-job interactions as opportunities to practice hearing ideas that differ from their own (78). Research on concepts such as psychological safety—a social condition in which team members feel included, safe to learn, safe to contribute, and safe to challenge ideas without fear of marginalization or retribution—also indicates that trust can be fostered through relational and technical strategies such as supporting bi-directional communication, increasing frequency of interactions, attaining quick wins, demonstrating empathy, inviting learning, and showing curiosity in the midst of failure (79, 80).

### Trust, mechanisms for change, and implementation outcomes

As mentioned previously, the theory of change integrates relational cohesion theory, relational cultural theory, and implementation research findings related to mechanisms of change (43). In doing so, the theory of change shows (1) how trusting relationships can change behavior in individuals and groups and (2) how these changes in behavior can contribute to use of evidence in health services and other related service delivery settings. Specifically, the theory of change captures how trust-based relationships exert influence on potential mechanisms of change that contribute to implementation outcomes. In this case, mechanisms are the processes through which trusting relationships contribute to commitment to and resilience for implementation efforts, and consequently, sustained implementation and evidence use.

Implementation team members provide a resource for change to happen and can support the achievement of implementation outcomes including sustained use of evidence. To fully understand how fostering trust among implementation team members can contribute to implementation outcomes, it is important to explore how trusting relationships can pull the necessary levers to facilitate behavior change among implementation stakeholders. Albers, Metz, and Burke (16) developed a logic model that hypothesized how implementation support activities may contribute to behavior changes leading to evidence use incorporating the Capability-Opportunity-Motivation-Behavior (COM-B) framework (53). The logic model informs the current theory of change, in which we describe how trusting relationships can yield the three pre-conditions for behavior change: (1) *capability*, representing information about changes in the physical, cognitive, or psychological abilities of implementation team members; (2) *opportunity* for changes in the physical, social, and cultural environments where implementation planning takes place that are prompted through trusting relationships; and (3) increased *motivation* in team members and implementation stakeholders to collaborate and achieve implementation outcomes.

The theory of change posits that trusting relationships contribute to changes in capability, opportunity, and motivation, eventually leading to improved implementation of evidence. Findings from a recent study (2) identified central themes in how ISPs approach their work, including supporting participatory learning and engaging in co-creation with implementation team members and stakeholders. Participatory and co-creative approaches are grounded in relational work and can contribute to changes in capability, motivation, and opportunity for evidence use. We outline these points in greater detail below.

#### Capability

*Building trusting relationships and supporting participatory learning can increase implementation team members and stakeholders' sense of capability*. As team members engage in peer-to-peer support and use data for decision-making they feel empowered and capable for supporting implementation efforts. Although it is important for team members to demonstrate capability, credibility, and “added value” to each other, it is through trusting relationships that implementation team members and stakeholders engage in the co-learning needed to increase their general sense of capability.

#### Motivation

*Building trusting relationships and engaging in co-creation can build intrinsic motivation for supporting implementation work*. Recent study findings (2) described the importance of authenticity and commitment in the work of implementation team members and stakeholders, both of which can motivate all stakeholders by validating their implementation efforts. Trusting relationships among team members and stakeholders promotes the risk-taking needed for the complex work of implementation.

#### Opportunity

*Implementation team members seek to build relationships among themselves and with additional stakeholders* (25). The trusting relationships among implementation team members often represent an additional layer of support for all stakeholders, creating the opportunity needed to attend to implementation challenges. Trusting relationships create the space needed for implementation teams to meet and reflect on implementation progress and serve as thought partners for the work. Trusting relationships *support communication, coordination, and collaboration*, which results in denser networks and closer relationships among stakeholders. These networks and relationships can provide increased opportunities for successful implementation of evidence.

Using the COM-B framework (53) and building on the work of Albers et al. (16), we posit that relational conditions such as trusting relationships enhance implementation capability, opportunity, and motivation, thereby enabling the concrete behavior change needed by team members to commit to implementation activities, continuously improve implementation efforts, and sustain use of evidence long enough to achieve population outcomes. These assumptions align with relational cohesion theory, which suggests positive exchanges among team members lead to higher levels of commitment and collectively-oriented behavior (48).

### Measurement

Taken together, the theory of change offers testable hypotheses that warrant ongoing empirical investigation, corroboration, and refinement. On this front, it will be critical to identify (or develop) and apply valid and reliable measures that capture information about key elements in the theory of change. Turning to the relational strategies outlined in the theory of change, existing scales, such as the Interpersonal Communication Assessment Scale [ICAS; (81)], possess items that tap into relevant interpersonal dynamics like high-quality communication and empathy-driven exchanges. The Individual Authenticity Measure at Work scale could also be adapted to tap into perceptions about vulnerability and authenticity of the self and others in the context of an implementation project (82). Co-learning as a relational strategy could be measured, at least in part, through use of the Self-Assessed Collaboration Skills (SACS) Instrument (83).

The technical strategies outlined in the theory of change lend themselves well to simple, investigator-developed items, particularly to measure the extent to which the attainment of quick wins is prioritized and realized, and the extent to which ISPs demonstrate relevant expertise for the project at hand. Team members could simply be asked to indicate the extent to which they agree with statements reflecting the presence of these technical strategies. Other strategies, such as frequent interactions and responsiveness, could also be measured using simple items and objectively quantified. For instance, the number of team interactions (e.g., meetings, convenings, check-in emails or phone calls) could be counted to gauge general frequency over time. Responsiveness could also be quantified in terms of how quickly, on average, ISPs provide requested materials and respond to email or other forms of communication throughout the duration of a particular implementation project.

With respect to constructs linking the relational and technical strategies with the cultivation of trusting relationships, some extant measures could prove useful. For one, there exist numerous measures of positive affective responses—such as the Positive and Negative Affect Schedule (PANAS), Subjective Happiness Scale, and Subjective Wellbeing (84)—that could be adapted to the particulars of a specific implementation project. Efforts to measure individual perceptions of the “value-add” of other team members could draw from the extensive education literature, which features relevant measures including various iterations of the Comprehensive Assessment of Team Member Effectiveness [CATME; (85)]. Researchers could also draw from observational and self-report measures of team psychological safety (86, 87) and existing measures of work predictability (88, 89).

There also exist relevant measures that aim to tap into various aspects of trusting relationships or relational cohesion. Some measures focus on the construct of interpersonal trust, enabling respondents to report on their own trustworthiness and the trustworthiness of a specific other [i.e., dyad-level trust; (90, 91)]. Some measures of trust also pertain to perceptions of trust that exist at the level of a team or group [e.g., Trusting Relationship Questionnaire; (92)]. Another family of measures relate to the construct of team cohesion, and seek to measure the nature of team relationships across several dimensions (93), including (a) task (i.e., bonding between group members that is based on a shared commitment to achieve the group's goals and objectives), (b) social (i.e., closeness and attraction within a group that is based on social relationships within the group), (c) belongingness (i.e., the degree to which members of a group are attracted to each other), (d) group pride (i.e., shared importance of being a member of the group), and (e) morale (i.e., degree of loyalty to fellow group members and willingness to endure hardship for the group).

In terms of the COM-B components in the theory of change, researchers could endeavor to identify, adapt, or develop suitable measures that match the context of a particular implementation project. Indeed, Howlett et al. (94) outline a process of mapping the particulars of a project to the COM-B components to inform the use of suitable measures—a process that could be replicated in research focused on implementation.

There is also a substantial body of literature offering suitable measures of work-related commitment [e.g., organizational commitment, occupational commitment, job involvement, work involvement, organizational withdrawal intention, occupational withdrawal intentions; (95, 96)] and work-related resilience (97). Drawing from this literature, there are promising opportunities to develop measures of resilience within the specific context of implementation work.

For cases in which measurement development is required, in-depth qualitative inquiry will be a valuable tool for mapping the conceptual landscape of some constructs outlined in the theory of change and with respect to a particular implementation project. Empirical work on this front could highlight common construct dimensions specific to implementation that could be targets for subsequent measurement development, yielding measures that could be applied in a multitude of contexts related to implementation work.

## Discussion

Suboptimal outcomes in implementation are due, in large part, to the dearth of tested theory in implementation science (51). As a consequence, implementation research has been limited in its ability to effectively inform implementation practice. In the case of trusting relationships, the field of implementation practice has lifted up the importance of trust in achieving implementation outcomes. Research findings from disciplines other than implementation science, such as psychology and social work, show robust evidence for the role of trust in supporting key aspects of implementation such as developing implementation teams, conducting continuous quality improvement cycles, and supporting effective feedback loops among stakeholder groups. To improve implementation efforts, the field needs testable theories that can generate empirical, context-specific findings. The proposed theory of change presented in this paper provides well-defined strategies for promoting trust, describes logical linkages between trusting relationships and mechanisms for creating behavior change, and identifies proximal and distal outcomes theorized to result in positive implementation outcomes.

Implementation science needs to create stronger connections between implementation research and implementation practice. Gaining greater clarity on how trusting relationships affect implementation processes and outcomes will enable improved communication and collaboration between implementation researchers and implementation practitioners. Although implementation frameworks offer a basic conceptual structure for understanding implementation constructs, testable theories are needed to create more generalizable knowledge for the field and to effectively inform implementation practice. Further, more information is needed on how implementation strategies are used in various contexts and the role of trusting relationships as a moderator of implementation strategies on implementation outcomes.

The proposed theory of change presented here is emergent and requires critical review, empirical substantiation, and refinement. The assumptions of this theoretical model are currently being empirically investigated. Research on this front aims to assess the feasibility of developing and delivering a training and coaching curriculum for implementation teams and stakeholders to build trusting relationships. This work will also assess whether building trusting relationships contributes to short-term outcomes such as trusting relationships among implementation team members; capability, opportunity, and motivation; and commitment and resilience for implementation. Empirical findings generated from this work can inform future studies on trust-building aiming to address questions related to how generalizable trust-building strategies are in different service contexts, how trust can be developed when engaging the voices of people and communities most affected by implementation decisions, and whether the complexity of implementation efforts shapes the selection and impact of trust-building strategies.

In order for the field of implementation science to be rigorous and relevant, we need testable causal models and a stronger connection between implementation research and implementation practice. The theory of change presented in this paper is an example of how we can develop testable ways of explaining phenomena such as trusting relationships by specifying plausible associations between implementation strategies, mechanisms for change, and outcomes. This theory of change also strengthens connections between implementation research and practice by articulating underlying assumptions related to how trusting relationships (what is emphasized by implementation practitioners) are related to behavior change and implementation outcomes (what is measured by implementation researchers).

## Data availability statement

The original contributions presented in the study are included in the article/supplementary material, further inquiries can be directed to the corresponding author.

## Author contributions

AM wrote the first draft of the paper. TJ drafted additional content for the paper. AF and AB reviewed, edited the paper, and providing additional text. LB and MV served as reviewers providing feedback. All authors contributed to the article and approved the submitted version.

## Funding

The development of this conceptual model is supported by the William T. Grant Foundation, Grant No. OR-202893.

## Conflict of interest

The authors declare that the research was conducted in the absence of any commercial or financial relationships that could be construed as a potential conflict of interest.

## Publisher's note

All claims expressed in this article are solely those of the authors and do not necessarily represent those of their affiliated organizations, or those of the publisher, the editors and the reviewers. Any product that may be evaluated in this article, or claim that may be made by its manufacturer, is not guaranteed or endorsed by the publisher.
